# Prevalence of Homicide-Suicide Incidents in Greece over 13 Years

**DOI:** 10.3390/ijerph19137736

**Published:** 2022-06-24

**Authors:** Maria Alexandri, Maria Tsellou, Anastasia Antoniou, Efstathios Skliros, Andreas Nikolaos Koukoulis, Flora Bacopoulou, Stavroula Papadodima

**Affiliations:** 1 Zagora Health Center, Magnesia, 370 01 Zagora, Greece; malexandri@gmail.com; 2 Department of Forensic Medicine and Toxicology, School of Medicine, National and Kapodistrian University of Athens, 115 27 Athens, Greece; maria_tse@windowslive.com (M.T.); stpapd@med.uoa.gr (S.P.); 3 2nd Department of Psychiatry, “Attikon” University General Hospital, School of Medicine, National and Kapodistrian University of Athens, 124 62 Athens, Greece; anastant@med.uoa.gr; 4 Nemea Health Center, Corinthia, 205 00 Nemea, Greece; stathis.skliros@gmail.com; 5 Department of Law, School of Law, Democritus University of Thrace, 691 00 Komotini, Greece; koukoulislawoffice@yahoo.gr; 6 Center for Adolescent Medicine and UNESCO Chair in Adolescence Health Care, First Department of Pediatrics, School of Medicine, National and Kapodistrian University of Athens, Aghia Sophia Children’s Hospital, 115 27 Athens, Greece

**Keywords:** homicide, suicide, Greece, firearms, intimate partner, familial, femicide, filicide

## Abstract

Prevalence of homicide-suicides is difficult to determine in Greece due to the lack of a national tracking system. The aim of this study was to estimate the prevalence of the homicide-suicide incidents in Greece over the past 13 years, and to determine the circumstances under which they occurred, as well as the characteristics of perpetrators and victims. Two Internet search engines (google and yahoo), as well as the search engine of the major national news websites, were surveyed to identify the number of homicide-suicide cases that occurred in Greece from January 2008 to December 2020. Over the study period, 36 homicide-suicide incidents occurred in Greece, resulting in 36 suicides and 41 homicides. The above incidents reflect an annual homicide-suicide rate of 0.02 cases per 100,000 inhabitants. Most perpetrators were male (88.9%), whereas most victims were female (80.6%). Spousal-consortial cases accounted for 52.7% and familial cases for 41.7% of the total number of incidents. The use of firearms (mostly shotgun) was the most common method of homicide and suicide (58.3% and 63.9%, respectively). Women killed only their children, while men committed homicide and suicide mainly in the context of a former or current intimate partnership with the victim. Our results are in line with international homicide-suicide data. The establishment of a national surveillance system for homicides-suicides would be of paramount importance as it would facilitate accurate recording, identification of risk factors and characteristics of potential victims and perpetrators and it could ultimately be an aid to the prevention of such tragic events.

## 1. Introduction

Homicide-suicide refers to a form of fatal violence during which an individual kills one or several victims, without their consent, and then, within a short period of time he/she commits suicide. For an incident to be defined as a homicide-suicide, the suicide must be temporally and motivationally related to the homicide [1,2].

Homicide-suicide incidents are tragic and devastating events that have a strong impact on the public. Most cases are characterized by a strictly spousal or intimate relationship between the perpetrator and the victim, although children may also be involved. Firearms are the lethal weapon in most instances [3,4,5]. Marital or extra-marital relationship dissolution, financial and health problems in combination with an apparent or latent hostility towards the victim may be the motives behind the act. Sometimes the events seem to have been the result of an outburst of violence originating from psychiatric disorder of the perpetrator in the context of a conflict [6].

Studies from several countries have shown rates ranging from 0.01 to 0.89 per 100,000 persons per year [5,6,7,8,9,10,11,12,13,14,15,16], whereas the percentage of homicide-suicide incidents in the total number of homicides has been reported to range from 4% in USA and Netherlands to 11% in Switzerland [17]. Various sources have been used to estimate the prevalence of homicide-suicide incidents, such as data from criminal registers, center for diseases control and prevention, police reports, forensic departments, news surveillance, etc. [18]. The largest of any existing data set regarding homicide-suicide cases is the Death National Violent Death Reporting System which is a state-based active web surveillance system across 3019 cities and 48 United States [19].

In Greece there is no national tracking system for the homicide-suicide incidents and the National (Hellenic) Statistical Authority records homicides and suicides separately [20]. To the best of our knowledge, there are no published studies regarding the prevalence of homicide-suicide cases among Greeks-residents in Greece. Therefore, the aim of this study was to use Internet search data query to estimate the prevalence of the homicide-suicide incidents in Greece over a period of 13 years (from January 2008 to December 2020), and to determine the circumstances under which they occurred as well as the characteristics of the perpetrators and victims.

## 2. Materials and Methods

Newspaper surveillance has been used in previous studies in USA [21,22,23,24], Italy [13] and Netherlands [25] to estimate the prevalence of the phenomenon. Two widely used Internet search engines in Greece and worldwide Google and Yahoo, as well as the search engine of the major national news websites, were surveyed to identify the number of cases of homicide-suicide occurring in Greece from January 2008 to December 2020. Several keywords in Greek and English language referring to homicide-suicide incidents were used (for example “homicide-suicide”, “he stabbed her and then he committed suicide”, “he shoot her and then he committed suicide”, “he stabbed her and then he shoot himself” and other similar expressions in several combinations)

For the purpose of this study, a homicide-suicide was classified as such if one or more person(s) killed one or more victim(s) and committed suicide within 1 week after the homicide. Cases of presumed double suicide (cases in which the victim agrees with the perpetrator’s intention) evidenced by a suicide note, were excluded.

Classification of homicide-suicide incidents was performed according to the typology described by Marzuk et al. [2]. This typology is based both on the relationship between the perpetrator and the victim and on the primary motive underlying the crime. A spousal relationship is defined as a current or previous relationship between the perpetrator and his/her partner. A consortial relationship includes current or separated intimate unmarried partners. When the perpetrator has killed multiple victims, the relationship with the primary victim is coded. The main types of homicide-suicide described by Marzuk et al. are: I. Spousal-consortial II. Familial (filicide-suicide, familicide-suicide) and III. Extrafamilial. The motives are classified as follows: A. Amorous jealousy, B. ‘‘Mercy killing”, C. ‘‘Altruistic or extended suicides’’, D. Family financial or social stressors, E. Retaliation, F. Other, G. Unspecified [2].

The data set collected included: (a) information about the murderer’s sex, age, marital status, (b) the relationship between the murderer and the victim (c) the method used for committing homicide and suicide; and (d) the motivation or reason that might explain the homicide-suicide as reported in news websites.

Statistical software IBM SPSS Statistics 25.0 was used for the statistical analyses. Sociodemographic characteristics of the sample were examined with descriptive statistics. Wilcoxon signed-rank test was performed for the comparison of the age between victims and perpetrators, while Fisher’s exact test was used for the comparison between male and female perpetrators regarding the method of homicide and suicide, motivation, location and their relationship with the victims. Correlation coefficient Pearson’s R was used in order to decide whether the ages of the victim and the perpetrator are correlated. Values with *p* < 0.05 were considered as statistically significant.

## 3. Results

During the 13-year-period (2008 to 2020), we retrieved 36 homicide-suicide incidents in Greece which resulted in 36 suicides and 41 homicides The above incidents reflect a homicide-suicide rate of 0.02 cases per year per 100,000 persons.

**Figure 1:**
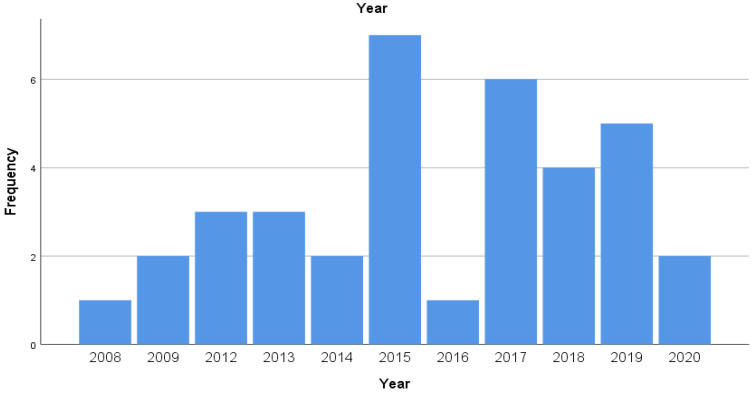
Number of homicide-suicide incidents per year from 2008 to 2020.

The number of homicide-suicide incidents per year ranged from 0 to 7 (mean value: 2.7 events per year), with no apparent trend over time (Figure 1). 

During the period 2008–2019 a total number of 5057 suicides and 1330 homicides were recorded by the Hellenic Statistical Authority (data for 2020 were not available). At the same period 34 homicide-suicide incidents occurred resulting in 34 suicides (0.67% of the total number of suicides) and 39 homicides (2.93% of the total number of homicides).

Analyses of the cases showed that in 3 out of the 36 cases (8.3%), the perpetrator killed more than one victim before committing suicide. In the first case a man killed his wife, daughter, and mother-in-law. In the second case a father killed his twin sons and in the third case a man killed his sister and her husband and son (brother-in-law and nephew).

The sex and the mean age of the perpetrators and the victims are shown in Table 1. The vast majority of the perpetrators were male (88.9%) whereas most victims were female (80.6%). The age of perpetrators ranged from 26 to 91 years, whereas the age of the victims ranged from 4 to 80 years. Twelve victims (29.3% of the victims) were under the age of 18 years. Wilcoxon singed-rank test showed that the perpetrators were statistically significantly older than their victims (victims’ age 39.56 ± 23.22 years vs. offenders’ age 53.03 ± 17.59). Moreover, Pearson’s R coefficient showed a correlation between the ages of the victim and the offender (R = 0.688, *p* < 0.001).

The most common method of suicide was the use of firearm (23 cases, 63.9% in the total number of cases). Shotguns had been used in 15 cases and handguns in 8 cases. Stabbing, hanging, poisoning, fall from height and fume inhalation after arson were also used by the perpetrators in order to commit suicide. Death of the victim was also often caused by firearms (23 victims out of 41, 56.1%), followed by stabbing (11 cases, 26.8%). Other causes of death were strangulation (2 victims, 4.9%), fall from height (2 victims, 4.9%), fume inhalation (2 victims, 4.9%) and drowning (1 victim, 2.4%). In Table 1 the percentages of perpetrators who used a particular method of homicide is shown (total number of cases:36). This is slightly different from the above percentages, given that three perpetrators killed more than one victim. Use of firearm was the method of homicide in 21 incidents, with the firearm being a shotgun in 17 incidents (81.0%).

The location of homicide and suicide was victims’ residence in most cases (69.4% and 66.7% respectively). In 33 out of 36 homicide-suicide incidents (91.7%) the location of homicide and suicide was the same. In two of the remaining cases the perpetrators were found dead in a rural area after killing their victims in their homes, and in the third one, the perpetrator committed suicide in his own home.

The most reported motive was family financial or social stressors (10 cases, 27.8%), followed by amorous jealousy (9 cases, 25.0%). The distribution of the cases according to the motive is shown in Table 1.

### 3.1. Spousal-Consortial -I (Intimate Partnership)

The analysis showed 19 cases of spousal-consortial (intimate partner) relationship, current or former. In 11 cases the perpetrator and the victim were married, in one case divorced (ex-spouse), in 5 cases unmarried intimate partners and in two cases former intimate partners. The perpetrators were all male. Their age ranged from 30–91 years and their mean age was 55.0 ± 16.3 years. The age of the victims-all female-ranged from 4 to 80 years. Their mean age was 40.1 ± 23.2 years.

In one case the man killed with a handgun his spouse, mother- in-law and 4-year-old daughter, before killing himself (the case was classified as spousal, because the spouse was considered as the main victim). In the rest of the cases the perpetrator killed only one person.

In one case the perpetrator killed his prior wife with whom he had been divorced 28 years ago. He had rent a house close to hers and he had dug a tunnel connecting both houses. He stabbed her multiple times. He was found dead several days later in a rural area.

Firearm injuries was the cause of death in the vast majority of homicide victims (17 out of 21, 80.9%) and suicide victims (16 out of 19, 84.2%). Other methods for committing homicide were stabbing (cause of death in 3 homicide victims) and strangulation (1 homicide victim). Other methods for committing suicide were stabbing (1 case), hanging (1 case) and poisoning (1 case). In 15 cases (78.9%) the method of homicide and suicide was the same.

Amorous jealousy (A) was the motive in almost half of the cases (9 out of 19, 47.4%). Three cases were considered as altruistic or “extended” suicides (C). They were three cases of elderly couples (males 91, 78 and 65 and females 80, 77 and 62 respectively) with health problems, mainly of the elder perpetrator. Frequent quarrels, categorized as family financial or social stressors (D) were recorded in 5 cases. In one case the motive was not derived from news reporting and consequently it was categorized as unspecified (G). In one case, the perpetrator and the victim had illegal drug use problems and the motive was categorized as “other” (F).

### 3.2. Familial-II

Fifteen (15) cases were classified as familial, including 15 perpetrators and 18 victims. In one case a 26-year-old man killed his half-sister with a shotgun and then killed himself in the same way motivated by his hostility towards his father. In 8 cases, parents killed their underage children and in one case a father killed his 45-year-old son. In 4 cases, the victim was the brother or sister and in one case the mother of the perpetrator. In 2 cases, there were more than one victim. More specifically, in the first case a man killed his sister, brother in-law and his nephew and in the second case a man killed his 8-year-old twin sons keeping them with him locked and setting their house on fire. The perpetrator and his sons died because of fume inhalation.

Most perpetrators were men (11 out of 15, 73.3%). Their age ranged from 26–86 years and mean age was 46.8 ± 13.1 years. Female victims presented a slight predominance (10 out of 18, 55.6%). The age of the victims ranged from 4 to 77 years and the mean age was 36.3 ± 22.7.

Most victims had been stabbed (8 out of 18, 44,4%), followed by 4 (22.2%) who had been killed by firearms. Other cause of homicide victim death was fall from height (2 victims), fume inhalation (2 victims), strangulation (1 victim) and drowning (1 victim). Regarding the death of the perpetrators, use of firearm was the most common method of suicide (5 out of 15, 33.3%), followed by stabbing (3 cases, 20.0%) and fall from height (3, 20.0%). Hanging (2 cases), poisoning (1 case) and fume inhalation (1 case) were other methods of suicide used by the perpetrators.

The main motives were family financial or social stressors (5 out of 15 cases, 33.3%) and “altruistic or extended suicide” (4 cases, 26.7%). Retaliation was responsible for 3 cases (20.0%), whereas in 3 cases the motive was unspecified. There was one case where the father killed his 18-year-old daughter because she had an intimate partnership and he considered that it was a dishonor to his family (“honor killing”). The father had a history of prior hospitalization in psychiatric clinic, and he was recently dismissed. The above case was classified in family financial or social stressors-problems.

There were two cases of filicide in which mothers throwed their daughters 4 and 5 years old and then jumped from height. The second case occurred only three months after the first one.

### 3.3. Extra-Familial

Only 2 extra-familial cases were recorded. In both the perpetrator and the victims were male and knew each other before the incident. In one case the victim was the perpetrator’s best man. The age of the perpetrators was 54 and 60 years and the age of the victims was 55 and 35 years. The motive in the above cases was recorded as unspecified.

### 3.4. Comparison between Male and Female Perpetrators

Fisher’s exact tests highlighted the differences between male and female perpetrators (Table 2). As far as the method for committing homicide and suicide, men preferred the use of a firearm. Regarding their relationship with the victim, women had killed only their children, while men committed homicide, mainly in the context of a current of former intimate partnership with the victim. No statistically significant differences were observed between sexes as far as motivation and location of crimes/suicides were concerned.

## 4. Discussion

During the past decade, Greece experienced a hard economic crisis. Because of its debt, a series of packages from the European Commission and European Central Bank and the International Monetary Fund were imposed, leading to the implementation of severe austerity starting in 2010. Several studies since then have reported an increase in suicides and suicide attempts, as well as anxiety and depressive disorders, especially among working-age men who were most affected by unemployment [26,27]. During 2014–15 the suicidal rates reached their zenith, although they were still the lowest in Europe. In 2015 a small decrease was observed, probably reflecting a small decrease in unemployment and a positive sign in the growth rate [28]. Homicide rates in Greece have also increased during the past decade. The homicide rate in 2009–2012 increased by 38% in comparison with the period 2005–2008. The largest increases were observed among the youngest (20–34 years) and the oldest (≥80 years) adults [29].

Homicides and suicides are officially recorded in Greece by the Hellenic (Greek) Statistical Authority (ELSTAT). However, there is no established system for recording homicide-suicide incidents. In order to overcome the above problem we used Internet search data query to identify them and to estimate their prevalence among Greeks and residents of Greece.

News surveillance has been considered as an important method of content analysis for the study of the phenomenon. Undoubtedly, there are several shortcomings to this type of data collection when trying to determine the incidence and prevalence of a condition in a population. The information in the newspapers or news websites depends on editorial policies and focuses more on public interest. This information is often incomplete and speculative, or even inaccurate, especially regarding the motivations of the murderer. Details about the psychiatric history of the perpetrator, as well as the dynamics of the environment, are usually not available. Important data from the autopsy report are also lacking, such as the toxicological results. The news may refer to the homicide more fully than the suicide, and even not mention the suicide if it is not contemporary with the homicide. Moreover, suicides that have been connected to homicides afterwards may not have been reported [21,22,23,24,25,26,27,28,29,30].

Regarding the accuracy of the method, Malphurs and Cohen [24] in the late 1990’s compared news reporting with the coroner’s reports in two Florida counties and found 71% of medical examiner–confirmed cases of homicide-suicide to be reported in the news media. The above portion is expected to have been increased during the following years, due to increased Internet access. Sallari and Sillito [21] examined state fatality reports in a western state of USA and found by internet search that every intimate partner homicide-suicide in the state fatality reports for the two years examined (2004–2005) was also reported in at least one local newspaper. Richards et al. [4] in their study about newspaper coverage of femicide-suicide cases from North Carolina during 2002–2006 found that approximately 16% of the cases could not be recovered from the media. Conclusively, according to the aforementioned studies, the accuracy of the method ranges from 71 to 100% depended on the characteristics of newspapers and news websites policies in every country.

Despite the limitations of a newspaper and/or news website review, it remains a useful tool to obtain data for the purpose of characterizing victims and perpetrators. News surveillance research has been used in many previous studies for examining homicide–suicide in general or among particular groups, such as between intimate partners or mothers and children with disabilities, in USA [22,24,31], Italy [13] and The Netherlands [25].

In a recent review of homicide-suicide studies, in 5 out of 49 studies included, the source of the data were newspapers, websearching or online forums [18]. In their review of 22 studies on intimate partner homicide-suicide, Zeppegno et al. report that medical examiners/forensic/legal medicine records had been used in 59%, newspapers/online archives in 41%), public security reports/court judgment records in 32% and specific datasets on domestic violence in 1% of the studies. In only a couple of studies information was retrieved from statistic institutes or census data. In several countries there is no standardized reporting system for HS, and consequently newspapers and online archives are the only way for identifying these incidents [32].

Homicide-suicide rate in our country was estimated to be 0.02 cases per year per 100,000 inhabitans. Epidemiological data from several countries have shown that homicide-suicide incidents are relatively rare, but substantial cross-national differences exist. Annual rates of homicide-suicide incidents have been described to be 0.23 per 100,000 inhabitants in USA [3], 0.08 in New Zealand [33], 0.89 in South Africa [5], but 0.01 in Ghana, West Africa [10], 0.18 in Hong Kong [34], 0.05 in United Kingdom [6], 0.17 in France [15], 0.04–0.06 in Italy [11,13], 0.2 in Finland [16], 0.05 in Sweden [9], 0.09–0.10 in Switzerland [12], 0.005–0.146 in Romania [8] and 0.2 in Croatia [14].

In our study, the number of homicide-suicide incidents per year ranged from 0 to 7 (mean value: 2.7 events per year), with no apparent trend over time. COVID-19 pandemic may have increased the risk for homicide-suicide incidents due to several factors, such as physical distancing, confinement measures, economical distress which resulted in increased rates of depression and anxiety. Contracting COVID-19 has also been reported as a risk factor due to panic and fear of stigmatization and isolation, driven by the misinformation, especially during the first period of COVID-19 pandemic [35,36,37]. We have not however observed any statistically significant increase in the number of homicide-suicide incidents during the year 2020. In our study the vast majority of homicide-suicide perpetrators were male (88.9%), whereas most victims were female (75.6%). Our findings are in accordance with previous studies from other countries [1,3,5,8,11,14].

Spousal-consortial cases represented 52.7% of our sample, which is accordance with previous studies from Italy [11], Australia [27], United States [3,25,38], Switzerland [1] and Hong-Kong [34] reporting that intimate femicide-suicide accounts for 43% to 61% of all homicide-suicide incidents. In the present study, all victims in spousal-consortial cases were female. Firearm injuries was the cause of death in 80.9% of the homicide victims and in 84.2% of the suicide victims-perpetrators. Amorous jealousy was the motive in almost half of the cases (47.4%), followed by family financial or social stressors (26.3%). Intimate femicide-suicide has consistently been reported as the most common type of homicide-suicide. Worldwide, the majority of the perpetrators in cases of murder-suicide are men and the victims are their intimate partners- most frequently women. Mostly, the act takes place in a domestic environment and involves the use of firearms [4,5,39]. The stress between intimate partners due to unemployment and domestic violence also plays a major role in the prevalence of homicidal-suicidal acts. The murder-suicide incidents among young adults are more likely to involve other victims such as children [21]. Especially in the cases where a man is the perpetrator, the act of homicide-suicide is characterized by feminists as an expression of “hegemonic masculinity” over their wife/partner and children.

In three elderly couples, the motive was health problems, mainly of the perpetrator. In this kind of cases, the perpetrators, probably thinking in an egoistic, self-centered way, believe that their spouse cannot suffer the difficulties of dealing with their health conditions (Alzheimer’s disease, cancer, etc.), but at the same time, they cannot continue to live without them. The suicide of the perpetrator in those cases seems to be the primary act and not the ultimate expression of remorse [40,41,42].

Familial cases also represented in our sample a considerable percentage of the total number of incidents (41.7%). Filicide-suicide incidents, which accounted for almost half of the familial cases (53.3%), have been connected mainly with altruistic and acute psychotic motives. The child is considered as the extension of the parent and both deaths represent an “extended” suicide [41,43]. Such ideations are frequently triggered by severe financial problems at home which cause major stress to parents who cannot meet their children’s needs. Another risk factor is depression of the parent which leads to suicidal acts. The depressed parents want to terminate their lives, but they do not want to leave their children behind. Thus, they decide to “mercifully” kill their children before committing suicide to protect them from potential suffering. In most cases, they choose to kill the children and themselves by painless methods, e.g., by carbon monoxide inhalation from charcoal burning, defenestration, etc., and then they usually leave a suicide note explaining the reasons for their macabre act [44,45,46,47]. It is interesting that these parents see their children as a part of themselves rather than an independent person with the right to live [47]. As far as the victims are concerned, girls and boys face the same risk of being the victims of filicide. Sometimes the arguments between parents could lead to extreme reactions such as filicide followed by suicide as an act of revenge against the spouse, especially by male perpetrators. In such cases, the suicide usually is not premeditated, but a panic reaction of the father to the hideous crime he just committed [46,48].

The suicide acts following homicides are usually performed in a 24-h time frame, and only in some rare cases, this frame may be extended to one week [3,4,12]. In almost all cases of our study, homicide and suicide occurred in the same day. Only in one case the interval between homicide and suicide was several days and the perpetrator was found in a remote rural area. However, probably only obvious cases of almost simultaneous homicide-suicide incidents are presented in mass media and consequently the exact number of incidents with a considerable interval between homicide and suicide may be unknown.

Studies from Italy [11], Turkey [41], USA [3,19], Belgium [42] and Germany [49] have shown firearms’ involvement in the majority of homicide-suicide incidents with percentages ranging from 57.0% to 96.3%. The greatest percentage (96.3%) has been observed in USA, probably because of the broad availability of firearms. Firearm use in homicide-suicide incidents has been reported to be less frequent (1.0–10.0%) in Hong-Kong [34], United Kingdom [6] and China [50]. Other methods of homicide described in the literature include strangulation, suffocation, stabbing, poisoning, and throwing from height, whereas falling from height has also been reported as a common method of suicide [16,32]. In our study firearms were used to commit homicide and suicide in 58.3% and 63.9% of the cases, respectively. In most cases a shotgun (long-barreled firearm) had been used, contrary to the existing data from other countries, in which handguns prevail in homicide-suicide incidents [10,12,23,27]. These findings are in accordance with a previous study from Greece regarding suicide by firearms [51].

Family financial or social stressors and amorous jealousy were the most common motives of homicide-suicide in our study (27.8% and 25.0%, respectively). Amorous jealousy as a motive has been discussed above. Regarding financial problems, Gregory and Milroy in their study in United Kingdom showed that 22.5% of the intimate partner offenders in homicide-suicide incidents were unemployed [52]. Similar percentages have been described by Chan et al. who reported that a quarter of the homicide-suicides in Hong Kong were triggered by economic disputes or problems, and more than one-third of perpetrators faced imminent economic crisis [34]. In a study from Turkey, 50% (5 out 10 cases) of the perpetrators had financial problems before committing homicide-suicide [41].

Our study has some limitations. It is likely that the above results underestimate the true incidence and prevalence of the phenomenon, and the small sample size may not be representative of the true demographics. National newspapers and news websites may report less than the true homicide-suicide cases. The motives recorded are only indicative, as they were concluded by the newspaper’s remarks, which may be oversimplified or even misleading, given that several aspects of the cases are revealed afterwards, during their thorough investigation by the Authorities. Information about the psychiatric history and the toxicological status of the perpetrators and the victims are also lacking. Further research should combine several methods, such as medical files and police files analysis, to estimate more accurately the annual incidence and nature of these events. It has been suggested that homicide-suicide incidents should be recorded into an International Classification of Diseases (ICD) code [24]. Moreover, the establishment of a national monitoring system for homicide-suicide incidents would be of paramount importance as it would facilitate accurate recording and identification of risk factors, as well as of the characteristics of the potential perpetrators and victims. On this basis, it would ultimately be an aid to the prevention of such tragic events [2].

Targeted prevention efforts should focus on maintaining a high level of awareness of the increased risk for homicide-suicide following situational stressors. The type of information that is important when determining who should be targeted for preventive interventions should include: (i) identifying potentially violent persons; (ii) identifying relationships with patterns of abusive behavior that could escalate to homicide; (iii) identifying communities less effective in executing protection orders to protect vicitms; and (iv) identifying communities in which access to mental health care and social services is difficult [13,27]. As firearm use is the most common method, firearm legislation restricting their availability could also be an important preventive measure [34,41,51].

## 5. Conclusions

Over the past 13 years, the annual homicide-suicide rate in Greece approximated 0.02 cases per year per 100,000 persons, in line with international homicide-suicide data. In most cases, perpetrators were male, there was one victim, a firearm (especially a shotgun), was used, whereas homicide and suicide were performed by the same method. Spousal-consortial cases (intimate partner relationship) showed a slight predominance over familial cases of homicide-suicide. Targeted efforts for identifying potential victims and perpetrators and maintaining awareness of the increased risk for homicide-suicide incidents in the context of circumstantial stressors, are of paramount importance for the effective prevention of this tragic phenomenon.

## Figures and Tables

**Table 1:** Sociodemographic characteristics of the study sample (*n* = 36).

Variables	Subgroups	Mean ± SD or *n* (%)
Gender of the perpetrator	Male	32 (88.9%)
Age of the perpetrator (years)		53.0 ± 17.6
Gender of the victim	Male	7 (19.4%)
Age of the victim (years)		39.6 ± 23.2
Relationship between offender and victim	Wife/girlfriend	16 (44.4%)
	Ex-wife/girlfriend	3 (8.3%)
	Sibling	5 (13.9%)
	Daughter/son	9 (25.0%)
	Parent	1 (2.8%)
	Extra-familiar	2 (5.6%)
Method of homicide	Shotgun/handgun	21 (58.3%)
	Stabbing	9 (25.0%)
	Strangulation	2 (5.6%)
	Drowning	1 (2.8%)
	Fall from height	2 (5.6%)
	Fume inhalation-arson	1 (2.8%)
Method of suicide	Shotgun/handgun	23 (63.9%)
	Stabbing	4 (11.1%)
	Fall	3 (8.3%)
	Fume inhalation-arson	1 (2.8%)
	Hanging	3 (8.3%)
	Poisoning	2 (5.6%)
Motivation	Amorous jealousy	9 (25.0%)
	Altruistic/extended suicides	7 (19.4%)
	Family financial/social stressors	10 (27.8%)
	Retaliation	3 (8.3%)
	Other	1 (2.8%)
	Unspecified	6 (16.7%)
Location of homicide	Home	25 (69.4%)
	Other	11 (30.6%)
Location of suicide	Home	24 (66.7%)
	Other	12 (33.3%)
Same location of homicide/suicide	Yes	33 (91.7%)

**Table 2:** Fisher’s exact test comparing relationships, method of homicide/suicide and motivation between male and female offenders (*n* = 36).

Variables	Subgroups	Sex		Fisher’s Exact Test	*p*
		Male (%)	Female (%)		
Relationship perpetrator-victim	Wife/girlfriend	16 (44.4%)	0	9.893	0.032
	Ex-wife/girlfriend	3 (8.3%)	0		
	Sibling	5 (13.9%)	0		
	Daughter/son	5 (13.9%)	4 (11.1%)		
	Parent	1 (2.8%)	0		
	Extra-familiar	2 (5.6%)	0		
Method of homicide	Shotgun/handgun	21 (58.3%)	0	16.914	0.001
	Stabbing	8 (22.2%)	1 (2.8%)		
	Strangulation	2 (5.6%)	0		
	Drowning	0	1 (2.8%)		
	Fall from height	0	2 (5.6%)		
	Fume inhalation-arson	1 (2.8%)	0		
Method of suicide	Shotgun/handgun	23 (63.9%)	0	13.357	0.011
	Stabbing	3 (8.3%)	1 (2.8%)		
	Fall	1 (2.8%)	2 (5.6%)		
	Fume inhalation-arson	1 (2.8%)	0		
	Hanging	2 (5.6%)	1 (2.8%)		
	Poisoning	2 (5.6%)	0		
Motivation	Amorous jealousy	9 (25%)	0	7.095	0.133
	Altruistic/extended suicides	5 (13.9%)	2 (5.6%)		
	Family financial/social problems	10 (27.8%)	0		
	Retaliation	3 (8.3%)	0		
	Other	1 (2.8%)	0		
	Unspecified	4 (11.1%)	2 (5.6%)		

## Data Availability

All data generated or analyzed during this study are included in this published article or are available from the corresponding author on reasonable request.

## References

[1] Shiferaw K., Burkhardt S., Lardi C., Mangin P., La Harpe R. (2010). A half century retrospective study of homicide-suicide in Geneva—Switzerland: 1956–2005. J. Forensic Leg. Med..

[2] Marzuk P.M., Tardif K., Hirsch C.S. (1992). The epidemiology of murder-suicide. JAMA.

[3] Regoeczi W.C., Gilson T. (2018). Homicide-Suicide in Cuyahoga County; Ohio; 1991–2016. J. Forensic Sci..

[4] Richards T.N., Gillespie L.K., Givens E.M. (2014). Reporting Femicide-Suicide in the News: The Current Utilization of Suicide Reporting Guidelines and Recommendations for the Future. J. Fam..

[5] Roberts K., Wassenaar D., Canetto S.S., Pillay A. (2010). Homicide-suicide in Durban; South Africa. J. Interpers. Violence.

[6] Flynn S., Gask L., Appleby L., Shaw J. (2016). Homicide-suicide and the role of mental disorder: A national consecutive case series. Soc. Psychiatry Psychiatr. Epidemiol..

[7] Regoeczi W.C., Granath S., Issa R., Gilson T., Sturup J. (2016). Comparing homicide-suicides in the United States and Sweden. J. Forensic Sci..

[8] Balica E., Stockl H. (2016). Homicide-suicides in Romania and the role of migration. Eur. J. Criminol..

[9] Sturup J., Caman S. (2015). Homicide-suicide offences: Description, classification and short case studies. J. Crim. Psychol..

[10] Adinkrah M. (2014). Intimate partner femicide-suicides in Ghana: Victims, offenders, and incident characteristics. Violence Against Wom..

[11] Verzeletti A., Russo M.C., De Ferrari F. (2014). Homicide-suicide in Brescia County (Northern Italy): A retrospective study from 1987 to 2012. J. Forensic Leg. Med..

[12] Panczak R., Zwahlen M., Spoerri A., Tal K., Killias M., Egger M. (2013). Swiss National Cohort. Incidence and risk factors of homicide-suicide in Swiss households: National Cohort study. PLoS ONE.

[13] Roma P., Spacca A., Pompili M., Lester D., Tatarelli R., Girardi P., Ferracuti S. (2012). The epidemiology of homicide-suicide in Italy: A newspaper study from 1985 to 2008. Forensic Sci. Int..

[14] Cengija M., Cuculic D., Petaros A., Sosa I., Bosnar A. (2012). Homicide-suicide events in Southwestern Croatia, 1986–2009. Med. Sci. Law.

[15] Saint-Martin P., Bouyssy M., O’Byrne P. (2008). Homicide-suicide in Tours, France (2000–2005)-description of 10 cases and a review of the literature. J. Forensic Leg. Med..

[16] Saleva O., Putkonen H., Kiviruusu O., Lönnqvist J. (2007). Homicide-suicide—an event hard to prevent and separate from homicide or suicide. Forensic Sci. Int..

[17] Liem M., Barber C., Markwalder N., Killias M., Nieuwbeerta P. (2011). Homicide-suicide and other violent deaths: An international comparison. Forensic Sci. Int..

[18] Rouchy E., Germanaud E., Garcia M., Michel G. (2020). Characteristics of homicide-suicide offenders: A systematic review. Aggress. Violent Behav..

[19] Fridel E.E., Zimmerman G.M. (2019). Examining homicide-suicide as a current in the stream analogy of lethal violence. Soc. Forces.

[20] Hellenic Statistical Authority ELSTAT. https://www.statistics.gr.

[21] Salari S., Silito C.L. (2016). Intimate partner homicide-suicide. Perpetrator primary intent across young; middle; and elder adult age categories. Aggress. Violent Behav..

[22] Coorg R., Tournay A. (2013). Filicide-suicide involving children with disabilities. J. Child Neurol..

[23] Lester D., Stack S., Schmidtke A., Schaller S., Müller I. (2005). Mass homicide and suicide deadliness and outcome. Crisis.

[24] Malphurs J.E., Cohen D. (2002). A newspaper surveillance study of homicide-suicide in the United States. Am. J. Forensic Med. Pathol..

[25] Liem M.C.A., Koenraadt F. (2007). Homicide-suicide in the Netherlands: A study of newspaper reports; 1992–2005. J. Forensic Psychiatry Psychol..

[26] Rachiotis G., Stuckler D., McKee M., Hadjichristodoulou C. (2015). What has happened to suicides during the Greek economic crisis? Findings from an ecological study of suicides and their determinants (2003–2012). BMJ Open..

[27] Konstantakopoulos G., Pikouli K., Ploumpidis D., Bougonikolou E., Kouyanou K., Nystazaki M., Economou M. (2019). The impact of unemployment on mental health examined in a community mental health unit during the recent financial crisis in Greece. Psychiatriki.

[28] Fountoulakis K.N. (2019). Suicide rate in Greece stabilizes at historically high levels but still lowest in Europe. J. Affect. Disord..

[29] Vrachnis N., Vlachadis N., Vlachadi M., Mastorakos G., Iliodromiti Z. (2015). Letter to the Editor—Homicides in Greece: Trends and Features. J. Forensic Sci..

[30] Genovesi A.L., Donaldson A.E., Morrison B.L., Olson L.M. (2010). Different perspectives: A comparison of newspaper articles to medical examiner data in the reporting of violent deaths. Accid. Anal. Prev..

[31] Warren-Gordon K., Byers B.D., Brodt S.J., Wartak M., Biskupski B. (2010). Murder followed by suicide: A newspaper surveillance study using the New York Times Index. J. Forensic Sci..

[32] Zeppegno P., Gramaglia C., Di Marco S., Guerriero C., Consol C., Loreti L., Martelli M., Marangon D., Carli V., Sarchiapone M. (2019). Intimate Partner Homicide Suicide: A Mini-Review of the Literature (2012–2018). Curr. Psychiatry Rep..

[33] Moskowitz A., Simpson A.I., McKenna B., Skipworth J., Barry-Walsh J. (2006). The role of mental illness in homicide-suicide in New Zealand, 1991–2000. J. Forensic Psychiatry Psychol..

[34] Chan C.Y., Beh S.L., Broadburst R.G. (2004). Homicide-suicide in Hong Kong; 1989–1998. Forensic Sci. Int..

[35] Ghossoub E., Wakim M.L., Khoury R. (2021). COVID-19 and the risk of homicide-suicide among older adults: Identify patients who are at risk and implement measures to protect them. Curr. Psychiatry.

[36] Mamun M.A., Bhuiyan A.I., Manzar M.D. (2020). The first COVID-19 infanticide-suicide case: Financial crisis and fear of COVID-19 infection are the causative factors. Asian J. Psychiatry.

[37] Mamun M.A. (2021). The first COVID-19 triadic (homicide!)-suicide pact: Do economic distress, disability, sickness, and treatment negligence matter?. Perspect. Psychiatr. Care.

[38] Comstock R.D., Mallonee S., Kruger E., Rayno K., Vance A., Jordan F. (2005). Epidemiology of homicide-suicide events: Oklahoma; 1994–2001. Am. J. Forensic Med. Pathol..

[39] Carretta C.M., Burgess A.W., Welner M. (2015). Gaps in Crisis Mental Health: Suicide and Homicide-Suicide. Arch. Psychiatr. Nurs..

[40] Bourget D., Gagné P., Whitehurst L. (2010). Domestic homicide and homicide-suicide: The older offender. J. Am. Acad. Psychiatry Law.

[41] Dogan K.H., Demirci S., Gunaydin G., Buken B. (2010). Homicide-suicide in Konya; Turkey between 2000 and 2007. J. Forensic Sci..

[42] De Koning E., Piette M.H. (2014). A retrospective study of murder–suicide at the Forensic Institute of Ghent University, Belgium: 1935–2010. Med. Sci. Law.

[43] Knoll J.L., Hatters-Friedman S. (2015). The Homicide-Suicide Phenomenon: Findings of Psychological Autopsies. J. Forensic Sci..

[44] Chiu N.M. (2010). Repeated filicide-suicide attempts by a mother with bipolar II depression. Prog. Neuropsychopharmacol. Biol. Psychiatry.

[45] Debowska A., Boduszek D., Dhingra K. (2015). Victim; perpetrator; and offense characteristics in filicide and filicide-suicide. Aggres. Violent Behav..

[46] Wei H.S., Chen J.K. (2014). Filicide-suicide ideation among Taiwanese parents with school-aged children: Prevalence and associated factors. Child Abuse Negl..

[47] D’Argenio A., Catania G., Marchetti M. (2013). Murder followed by suicide: Filicide-suicide mothers in Italy from 1992 to 2010. J. Forensic Sci..

[48] Carruthers G. (2016). Making sense of spousal revenge filicide. Aggres. Violent Behav..

[49] Siems A., Flaig B., Ackermann H., Verhoff M.A., Parzeller M. (2017). Homicide-suicide. Rechtsmedizin.

[50] Densley J.A., Hilal S.M., Li S.D., Tang W. (2017). Homicide–Suicide in China: An Exploratory Study of Characteristics and Types. Asian J. Criminol..

[51] Kastanaki A.E., Kranioti E.F., Papavdi A., Theodorakis P.N., Michalodimitrakis M. (2010). Suicide by firearms on the island of Crete: A 9-year record. Crisis.

[52] Gregory M.J., Milroy C.M. (2010). Homicide and suicide in Yorkshire and the Humber: 1975–1992 and 1993–2007. Am. J. Forensic Med. Pathol..

